# An Essay on the State of Medicine, and on the Prevailing Diseases of European and Asiatic Turkey

**Published:** 1837-10

**Authors:** 


### ART. IV.

*Ueber den Zustand der Heilkunde und ueber die Volkskrankheiten in der Europäischen und Asiatischen Türkei. Ein Beitrag, &c.* Von FRIEDR. WILHELM OPPENHEIM, Doctor der Medicin und Chirurgie, &c.—*Hamburg*, 1833. 8vo. pp. 143.

*An Essay on the State of Medicine, and on the prevailing Diseases of European and Asiatic Turkey.* By F. W. OPPENHEIM, M.D.—*Hamburg*, 1833.

AFTER we had transmitted to a friend at Constantinople a series of queries respecting the present state of medicine in Turkey, with a view to obtaining a Report on that subject, for insertion in the Fifth Part of some of our subsequent Numbers, we had the pleasure of receiving from the author the very valuable and interesting work now before us, of the existence of which we were previously ignorant, and which more than supplies the information we had sought from our own correspondent. In the present article, we shall endeavour to lay before our readers a comprehensive but brief analysis of the more important matters contained in Dr. Oppenheim's little work, adopting our own arrangement, and breaking the thread of the narrative with but few observations or critical remarks of our own. We must, however, commence by stating our conviction, founded on a careful perusal of the book, that, exclusively of its medical details, it contains more new and accurate information respecting the character, manners, and customs of the Turks, than any of the numerous and large works—we had almost said, than all of these—which have been published during the last twenty years. This arises from the peculiar advantages enjoyed by Dr. Oppenheim derived from his long stay in this country, and the unequalled opportunities which his particular position afforded him of seeing the natives more closely and more at home, than can be done by the mere traveller. It would appear that Dr. Oppenheim entered Turkey during the last Russian campaign, in the medical department of the invading army, and that, after the peace, he passed into the Turkish service, under the auspices of the Grand Vizier.

"A residence of almost three years in the different provinces of Turkey, both in Europe and Asia, has given me full insight into the manners and customs of the people, as well as made me acquainted with the state of medicine among them; and this chiefly because I did not spend the greater part of this time in the capital, as was the case with most physicians who have given us any accounts of these countries, but travelled through the various provinces in my capacity of an army medical officer. And further, in the latter part of my stay, through the particular introduction of the Grand Vizier and other Turks of eminence, I had access to the Turks themselves; a thing so difficultly obtained by a foreigner: after I had made myself master of the customs and the language, and had adopted the oriental dress, I was treated, not as a Frank, but as a true believer, and received freely into their families." (*Preface*, p. vi.)

The knowledge of medicine, in all its branches and in all its foundations, is at the lowest ebb among the Turks; so low is it, that it requires some effort to comprehend how it can happen that a people, so long possessing a fixed government and civil institutions, and in constant relation with polished nations, could remain to this day almost as destitute of medical and of all kinds of scientific knowledge as the savages in the South Sea islands. Yet such is nearly the case. The nature of our work will not permit us to enter upon the consideration of the causes of this singular result, but we may state that it mainly flows from the nature of the Mahomedan religion, and the modes of thinking and acting to which it has given rise. It is sufficiently apparent from Dr. Oppenheim's work that, although some slight advances have certainly been made, and are making, towards a more rational system of medicine, through the residence of foreign physicians, now daily more countenanced by the government, still it is impossible that medicine can flourish while Islamism is, as at present, not merely the national faith, but the deeply-cherished and enthusiastic love of the whole nation. It is a melancholy truth that the whole mass of the Turkish people are, in respect of the cultivation of the intellect, still barbarians; and it is more melancholy still to know—and no reader of the work before us can fail to know,—that the state of moral cultivation is, if possible, even lower than that of the intellectual. Animal gratification of the grossest kind is the governing motive of the whole male population; and, perhaps, the most striking illustration that could be adduced, at once, of the degradation of morals and of medicine in this unhappy country, is the fact, demonstrated by every page of the work of Dr. Oppenheim, that the most prized, if not the principal, part of the miserable duties of the native doctors, is to minister, by fancied restoratives and corroborants, to the palled appetites and jaded powers of an exhausted or superannuated sensuality. It is impossible to contemplate the dreadful consequences flowing directly and unquestionably from the doctrines of Islamism, without admitting that Christianity, in its most corrupted and degraded form, has claims to the respect and patronage of the philosopher and philanthropist, whether a believer or an infidel, of an immeasurably superior kind to those which can be preferred by the religion of Mahomet, even in its purest and most perfect condition: and no human being, with an intellect above a savage, can fail to express his satisfaction, in perusing the degrading details here presented to him, that his lot has been cast in a Christian land, amid the blessings of civilization and the institutions of a free state. It remains to be seen to what extent the example of the present ruler of Egypt will operate upon the Turkish empire at large. It is certainly a most important step gained that, under his authority, both the principles and practice of European medicine and surgery have become triumphant, not only over the ignorance, but over the prejudices and religious scruples of the people. It is also a most fortunate circumstance, that in the present head of the medical institutions of Egypt, Clot-Bey, great knowledge, great skill, and great courage are combined. In spite of one of the most inveterate of the popular prejudices, the inviolability of the dead, anatomical dissections are now publicly practised in Egypt, under the superintendence of this distinguished man, while the plague itself is losing half its terrors, and probably will speedily abate its ravages, under the influence of his enlightened courage.

The practitioners of medicine in Turkey are of various kinds and orders,—Turks, Greeks, Jews, and Franks or Europeans. The native, or Turkish doctors, are to a man ignorant of the first principles of medical science, and the slaves of the most blind empiricism or grossest superstition. Anatomy is totally unknown and unpractised, and must be so while the existing religion is strictly maintained. To the Turks, however regardless of life, every dead body is sacred. The opening of dead bodies is expressly forbid by the Koran, "even should the dead person have swallowed the most costly pearl, which did not belong to him." There is admitted no exception to this, except in the case of a pregnant woman dying while the child gives signs of life; in which case the Cæsarean operation is permitted. The present Sultan, it is true, has had published, by special command, a large work on anatomy and medicine, containing numerous anatomical plates; but we are told by Dr. Oppenheim, that even this imperfect substitute for dissections is not known to a single practitioner in the empire, except the immediate pupils of the school recently established in Constantinople. It is somewhat singular that, although the pulse is regarded by the Turks as capable of indicating everything necessary in diagnosis and practice, they are altogether ignorant of the circulation of the blood. Divination is still one of the principal agents in the medical practice of Turkey; the influence of the stars, necromancy, talismans, amulets, and cabalistic figures, are the objects of their undoubting faith. Sometimes disease is regarded as an evil spirit, and is attempted to be conjured away; at other times it is considered as an immediate judgment from God, whose wrath is to be appeased by long prayers and the counting of the rosary: at one time a passage from the Koran is written on paper, pounded into a bolus and swallowed, or the words are written on a board, washed off, and the inky water given as a draught. Amulets, both as prophylactics and means of cure, are in the highest estimation, and indeed by all classes of Orientals, as well Christians and Jews as Turks. They mostly consist of triangular pieces of paper, containing written passages from the Koran or Bible, sewed into some part of the dress. These are deemed also very important in protecting the wearers from the *evil eye ;* a superstition of universal belief in Turkey. The doctors religiously observe also what they term their white and black, or lucky and unlucky days: on the last, no course of treatment is undertaken, no surgical operation performed.

Next in number to the native doctors are the Greek practitioners, who may be divided into three classes,—those who have studied medicine in Europe, generally in Italy, for a short time; those who have inherited their knowledge from their fathers; and, lastly, those who have been servants, assistants, or apothecaries for a certain time, with some so-called doctor. Of these three classes, the most ignorant and dangerous, and of course most pretending, are the first. The dangerous thing of a little learning was never better illustrated than in them. The oldest members of this fraternity are stanch Brunonians, and deal in nothing but bark, camphor, valerian, and such like; the juniors are all disciples of Rasori and Broussais, and wield their depletory arms, leeches, venesection, emetics, &c. as boldly and indiscriminately as the former do their nervines and their stimulants. The hereditary doctors come chiefly from a particular district in Lower Albania; they have never studied medicine at all, and practise purely as empirics, on the stock of recipes and specifics transmitted as heir-looms from father to son. These, as well as the third class, retain their national costume; the first, on the strength of their European education, and because it confers some fiscal privileges, wear the Frankish dress.

In the same rank of knowledge and consideration as these last are the Jewish doctors, who generally keep shops in the bazaars and hawk about their drugs and their medical skill, their cosmetics, and their dye-stuffs, to all comers. When summoned by a passing patient, they ask no questions, but feeling the pulse with an "I know your disease," administer on the spot a pill or powder, receive their fee of a couple of paras,* and recommence their cry "Ei Hekim," (good physic!)

It is not to be much wondered at, amid such a degradation of native science and skill, that the Turks should be disposed to think highly of European physicians. Indeed, such is their ignorance and faith in this matter, that every individual that appears among them in the European dress is not only regarded as a doctor, but is often *constrained* to act as such. *Frank* and *physician* are almost synonymous terms; and to every one that wears a hat, a hundred arms are outstretched wherever he goes, with a request to feel the pulse, the universally believed indication of all diseases and their cure. With this prejudice in favour of Foreign practitioners, it will readily be believed that Frankish adventurers of all sorts, with or without medical knowledge, literally

> Ambubaiarum collegia, pharmacopolæ

> Mendici, mimæ, balatrones, hoc genus omne—

are not wanting to take advantage of the national credulity, and to profess and practise an art of which they know absolutely nothing. Of the regimental surgeons mentioned by Dr. Oppenheim, one had been a letter-

* A marvellously small fee, since there are twenty paras in a German groschen and about eight groschen in a shilling.—Rev.

writer in Corfu, another a captain of a Ragusan trader, who took to physic when he had lost his ship. A non-commissioned officer in the army of Piedmont, who had taught French in the Consul's family at Adrianople, took it into his head to turn doctor; and, through the strong power of his own will, actually became so. Furnished by Dr. Oppenheim with *sixteen recipes,* he was forthwith installed as body physician to the Aga of Iambul. On another occasion, in Smyrna, our author was invited to meet in consultation with a French *physician,* who had been drum-major in the army of Napoleon!

After this account of the generally degraded state of medicine and its professors in Turkey, it is but justice to our author, and to the gentlemen commemorated by him, to state that there are to be found in various parts of the empire some Greek physicians who have received an excellent education in Europe, and who practise their profession with the skill of men of science and the dignity of gentlemen; but alas, their numbers are extremely limited.

With the exception of Constantinople, there are no apothecaries' shops properly so called, in any part of Turkey; nor is this to be expected in a country where the great majority of the practitioners cannot read, much less write a receipt. Of course, there is no national pharmacopœia, and every one prescribes and administers what seems to him good.

With all their ignorance, the professional men in Turkey are most highly esteemed. *Omne ignotum pro magnifico:* and the universal ignorance of the nature and powers of the healing art among this people, gives it, in their imagination, a value which it never can possess. Physicians, or persons so called, are universally regarded as in some degree sacred, and the title of doctor (*Hakim*) is the surest protection against all kinds of political and religious persecutions. Even the Greeks give to them the title of *Excellency* (εξοχοτατος). Nevertheless, it would appear, from Dr. Oppenheim's account, that professional services are not very liberally rewarded, at least those of the native doctors.

"The sick Turk promises much, but the cured one pays little. (*Medicis in morbis, &c.*) He rarely pays for any thing more than the medicine; and, as the physician most generally makes that up himself, he regulates his charges accordingly. If the patient dies, there is but little chance of the physician receiving any thing for his trouble; and, if he recovers, he soon forgets both disease and doctor. These remarks, however, apply only to the native practitioners; the Franc or European physician is almost always adequately remunerated, though the Turk does not reward the skill of the physician, but only pays for the actual labour bestowed on him. This is sufficiently indicated by the name given to the physician's honorarium, (Ajakderesi, *foot-money,*) which is put into his hand on his departure by the patient's servant, and amounts generally to a half or a whole *marmudié,* a gold coin worth from twenty to forty Turkish piasters, and equal to from two to four of our German dollars, [from five to twelve shillings, English;] besides this, the physician's servant, in most cases, receives as *bakschisch* (drinking money,) a *barbut,* the least Turkish gold coin, and equal to about six German groschen [about one shilling, English.] When these Ajakderesi are not tendered on the first or second visit, the physician does not repeat the visit till his fees have been sent him, and he is again invited to renew his attendance. In many cases, also, the attendance is not paid for till after the cure, which is particularly the case in attendance at the harem.

"In important cases, particularly in such as require energetic measures, or in cases of surgical operations, specific bargains are made, and these are sometimes settled in presence of the cadi. In cases of this kind, the physician engages to cure the patient within a given time, for a stated sum to be paid to him after the recovery. In these cases, the Turks are constantly cheated by the Greeks, as they refuse to delay the payment till after the cure, and insist on receiving a third or even the half of the stipulated sum in advance, well knowing that the chances are, not that the patient will recover, but that he will die, and that the doctor will then lose any unpaid part of the sum agreed on.

"The most effective method for the physician to secure himself against fraud, is to make his bargain in the presence of the Cadi; for, as this latter is entitled by law to ten per cent. of the sum agreed on, he invariably decides in favour of the physician, unless indeed the patient offer him more than the ten per cent. he is legally entitled to; in which case, venal justice will most certainly preponderate in the opposite scale.

"Payment for medical attendance is not always made in money, but is sometimes tendered in the shape of provisions, slaves, horses, arms, &c. I had cured the governor of Adrianople of a long standing fever; after his recovery, he asked me if I was married; on my replying in the negative, he offered me, as a present, two beautiful Greek slaves; and, when I declined his offer, stating that no women were allowed in the train of the army, he wished to give me a boy, and was much astonished at my refusal of so valuable a present and want of gratitude for his liberality. In a little town in Asia Minor, called Balükassar, I had cured the Aja of a large nasal polypus; his expressions of gratitude for the service rendered him were very warm; 'but,' added he, 'I have no money to pay thee with; take, therefore, this Curdish stallion and the camels which shall accompany thee as far as Konia.' Accordingly, when I took my leave, I found in my train two camels loaded with sheep and goat cheese, which he had sent in lieu of money.

"In cases of operation, it is still more advisable that the physician should settle the terms in presence of the judge of the place; not merely to secure the payment of the sum agreed on, but also to protect himself against private revenge. On such occasions, the practice is for the patient, or one of his relatives, to accompany the medical practitioner to the Cadi; or, in large towns, where such a functionary is to be found, to the Mufti, who draws up a *fetiva*, by which the practitioner is exonerated from all blame and responsibility in case of a fatal result, and has secured to him a stipulated but generally inadequate sum, amounting on the average to from one hundred to two hundred piasters, (from twenty-five to fifty German mark.) The operation for the stone forms, however, an exception to this general statement, as it is satisfactorily compensated. In cases of failure, it is usual to pay only half the sum agreed on.

"But the case is very different if the physician should have the misfortune to lose a patient, not in consequence of a surgical operation, but of some internal disease; for, in such a case, he runs great risk of answering with his own for the life of the deceased, and more especially if he happened to be connected with the diplomacy of the country, or was invested with some high office. In a case of this kind, the relatives, exasperated by the loss of the property of the deceased, which is generally forfeited to the imperial treasury, will seek to avenge themselves on the person of the physician. Under other circumstances, the relatives are easily appeased with the remark that fate had fixed that hour for the death of the deceased, and, as he was gone to Paradise, death could not be considered as a misfortune for him." (P. 20.)

The intercourse of physicians with the female part of the population, when sick, is, of course, remarkably restricted by the national mode of treating women. The following account of Dr. Oppenheim's first visit to the harem is interesting to us chiefly in a medical point of view, as affording another striking illustration of the barriers thrown by the Mahommedan religion against the progress of medical science, or rather against the beneficial operation of medicine as a practical art. It is, of course, impossible that any disease can be properly treated in the harem, —in other words, any disease of the female sex,—whilst such regulations are enforced as are noticed in the following extract:

"Like every one else, I was extremely anxious to judge from experience of the beauty of the Circassian and Georgian women, who are brought in their earliest youth to Constantinople to be sold, and thence sent into every part of the sultan's dominions, either to perform the menial offices in the harems, or to bear children to their lords. I was also very desirous to see the interior arrangement and management of these female colonies; and fortune soon offered me an opportunity of satisfying my curiosity. The favorite wife of the Kiaja-Bey (commercial agent) of the governor of Adrianople had been sick for three days, and the Pasha, who placed implicit confidence in me, declared I could most certainly cure her, if permitted to see her. The Kiaja-Bey, to whom I was not personally known, sent to request me to accompany his Harem Kiaja, a black eunuch, to his harem, which lay at more than a quarter of a league from his house. We proceeded to a low door, which was opened on our knocking, and were admitted into a garden: here I found an airy pavilion, the coolness of which was preserved by a magnificent fountain and cascades. In this delightful spot I was invited to rest, and served with coffee and a pipe, while my arrival was announced. After a delay of a quarter of an hour, I was conducted through the garden to a second door, where I was received by a veiled woman, the superintendent or portress of the harem, who likewise conducted me through a garden into the building appropriated exclusively to the use of the women; when a number of slaves and children, white and black, crowded round me with eager curiosity, or peeped from behind the curtains. At last the sick chamber was opened to me; a neat little apartment with red furniture and closed curtains. The fair patient was lying on cushions arranged on the carpetted floor, close to an ottoman, and covered from head to foot with a white cloth, in such a manner as to leave the beholder in actual doubt of her presence. I was directed to take a seat on the ottoman nearest to the head of the couch, and all the curious attendants were dismissed, leaving in the apartment, besides myself and interpreter, only the two children of the sick lady, of four and five years of age, and an old nurse. The patient answered my questions through the veil, without hesitation or prudery; even such as would have been considered by young ladies in Europe as not very agreeable. When I expressed a desire to feel her pulse, two pretty white hands were protruded from under the covering; and, when I asked to see her tongue, the patient slightly raised her veil, yet in such a manner as to allow me to obtain a glance of the features of a most lovely brunette, that could scarcely have reached her twentieth year. She, however, instantly after shrunk back under the drapery, like a snail into its shell, and requested I would now leave the room, and address any further questions to the nurse, who was well acquainted with her state. I was consequently conducted by the nurse into the *selamlick*, the antichamber of the master, and I was again treated with coffee and a pipe.*

"I was ultimately conducted into the presence of the Kiaja-Bey, who questioned me respecting his wife's state of health, and wished to know when she would be perfectly recovered, and if it would be necessary that I should visit her again? I answered, that the latter would not be necessary, and that the lady would be perfectly well in a few days, if the directions I had given were attended to. He testified his satisfaction with these answers by a motion of his head, and, after the honorary ceremony of coffee and pipes had once more been complied with, he directed the *hasnadar* (treasurer) to hand over to me a purse of five hundred piastres. My prognosis was verified: on the third day the lady was perfectly

* "I was much satisfied with this my first visit to a Turkish harem, and expected to meet with the same reception on every occasion; but I had knocked, on my first excursion, at the most liberal door, for I seldom after met with such easy access."

recovered, and my reputation rose with all classes to the highest degree of confidence." (P.34.)

"After a physician has visited the inmate of a harem, the usage of the country requires that he should send every morning to the master, to enquire how the *channem* (the gracious lady) has slept, and finds herself. This message is forwarded by the master to the invalid, in the name of the physician; and the lady returns her answer with thanks, and mentions at the same time when and at what hour she wishes to see him again. This ceremony is continued till the physician receives from the harem a silk shirt, a pair of silk drawers, an embroidered sash, an embroidered handkerchief, and a pair of embroidered socks. This is a token of thanks from the lady to the physician for her cure. The medicines are paid for by the husband at a later period, as well as the attendance, in case the fees have not been paid at the time of visiting." (P. 40.)

A striking mark of the general ignorance of the people concerning medicine, and of the ignorance and baseness of its ordinary professors, is furnished by the extraordinary value attached to the pulse as a means of diagnosis and prognosis.

"Nothing can give the doctor a higher place in the estimation of his patient than his drawing from the state of the pulse alone the whole of his judgments respecting the state of the malady. By means of it, he must not only know the complaint, but also whether and how the patient has slept; if he has taken any thing, and what this may be; the nature of the evacuations, &c. Every question the patient has to answer is painful to him, and with every query the estimation in which the physician was previously held obviously diminishes. At the very first visit the physician is expected to indicate, merely by feeling the pulse, the minute at which death will ensue, or when a favorable crisis will relieve the patient from all his sufferings. Nor is the opinion that feeling the pulse is the only thing necessary to the physician confined to the lower order, but is very prevalent among the Turks even of the highest rank. Of this the governor of Adrianople, Halilsh Pasha, afforded me a striking proof. On a visit to the Russian camp, after the peace, he entered the tent of General Paulin, attended by a numerous suite. Among the persons assembled were two other physicians, besides myself. Immediately after we had been introduced in this capacity to the pasha, he extended his arm to each of us in turn, and requested we would feel his pulse: this being complied with, he turned to the general, and pointed out to him the one he considered the best physician; this, he said, he had discovered from his manner of feeling the pulse, and wished them to make arrangements with him to enter into his service. This leads the cunning Greek practitioner to ascertain from the servants, in an indirect manner, the state of the patient, whose confidence is thus gained, and whose credulity is so much abused as to make him believe that the practitioner can, by feeling his pulse, see into the inmost recesses of his body." (P. 15.)

It is unnecessary to say that, in such a condition of the knowledge of medicine, its practice must be miserably defective, even in the hands of those who give the preference to ordinary medical measures over the weapons of superstition. Like the ignorant in other countries, the Turk, when he takes a *bona fide* medicine, loves to see some positive physical effect from it; and the larger the dose administered, he is, in general, the better satisfied. Emetics, however, are not approved of, and enemata are totally refused. It is customary, in the month of May, to undergo a course of medical treatment. This is usually commenced with a purgative, which is followed up for three or four weeks by tisans of the juices of different roots, as taraxacum, also by potions of whey, and, above all, by viper-broth, which the Turkish doctors regard as the best of all purifiers of the blood. The following account of a *consultation* between our author and a Persian physician may amuse our readers, and at the same time will give them some insight into the state of anatomical and therapeutical science in the East.

"At Magnesia, in Anatolia, I was requested to visit a distinguished Uelema, who was suffering from a decided hepatitis. I immediately opened a vein, and gave him every two hours two grains of calomel, which I had with me. The medicine given to him by the Persian doctor consisted of large jars of decoctions of plants, as the sickness, according to his decision, proceeded from a drying and consuming of the entrails. On the following day, shortly before my departure, I accidentally met the Persian physician at the bedside of the patient. He was a tall, spare man, with black hair and beard, an austere eye, and a fanatical look, which bespoke his hate and contempt for the unbeliever. It appeared to cost him a great effort to address me: at last, when I had asked the patient how he was, and he had admitted that he felt better after having taken my powder, the doctor said to me, 'Doest thou dare to say that his bowels are not diseased?' To this I assented. 'In what, then, are contained the bowels in the belly?'—'In a sac, in a skin.'—'It is false! They swim in a lake or pool: in this case the pool is dried up, and the entrails are nearly on fire: hence the intense thirst, the heat, the pain in the body, the dry tongue and skin, and scanty discharge of urine; and therefore thou hast dealt unjustly towards him, in depriving him of fluid blood, and in giving him solid instead of fluid medicines. To inflammation thou shouldst oppose moisture; to dropsy, dry remedies; heat should be expelled with cold, and cold with heat.'" (P. 68.)

A really valuable part of Turkish medicine, however, is the bath, the general use of which, with its accompanying operations of friction, stretching, pinching, and all the other manipulations of *massing* and *shampooing*, is of the highest benefit in the prevention and treatment of many diseases, more particularly in a country where the more ordinary mode of cure by internal remedies is almost always useless, if not injurious. The following is Dr. Oppenheim's account of the bath and mode of bathing.

"The public baths are beautiful stone buildings, covered with domes, and of elegant architecture; several of which are found in every town. They consist of large apartments, with marble floors. At the entrance there is a roomy hall, lighted from the roof, and having a large basin and fountain in the middle. Along the walls of this hall are ranged high and broad benches, covered with mattresses and cushions; and here the bather undresses, binds a silken cloth round his body, and puts on a pair of wooden sandals before he proceeds to the bathing rooms. The first of these is but moderately heated, and is intended to prepare the bather for the heat of the inner vaulted room (about 100° Fahrenheit,) which likewise receives light from the cupola. In the middle of this last-mentioned apartment there is a large square bench, raised a few inches above the floor, on which the bather reclines, while an attendant kneads for a considerable time every part of his body. After this operation, the bather rests for some time in this or in one of the adjoining recesses: these have a heated marble pavement, and large marble basins to receive the hot water from pipes fixed in the walls: properly speaking, this is the bathing apartment. After the bather has sufficiently recovered from the kneading operation, he stretches himself naked on the heated pavement, and his whole body is rubbed and washed with a piece of horse or camel-hair cloth, and the lather of scented soaps, and then washed clean with hot water; he is afterwards dried, and both head and body are wrapped in warm cotton clothes, and in this attire he returns to the first or outer hall, where he rests for half an hour on one of the couches, takes a cup of coffee or a glass of sherbet, and smokes a pipe: he then dresses, and returns home. The cost of this bath is very trifling, and must be so, since it is within the means of the lower order of the Turks, who use it frequently. The baths for the women are everywhere separated from those used by the other sex." (P. 18.)

As in some of the former instances, we find at once the miserable condition of medical knowledge and of morality strikingly illustrated by the account given, by Dr. Oppenheim, of the frequency of poisoning in Turkey, as well from ignorance as intention.

"As there is no medical police whatever in Turkey, the sale of poisons is not forbidden, and fatal cases of poisoning, arising out of the ignorance of the purchaser as well as seller, are not at all uncommon. The doctors who keep medicaments for sale give to every applicant whatever he asks for, and in any quantity. Sugar, salts, arsenic, &c. are heaped together in open chests and baskets. Arsenic is not only sold in the apothecaries' shops to any one who may apply for it, but the next article called for is weighed in the same scales, to which, perhaps, adheres poison enough to send the purchaser into the other world. When called into one of these wretches, made patients by their criminal negligence, the doctors, instead of feeling horror at the dreadful cramps and convulsions, unconcernedly shrug their shoulders, and leave him to his fate, declaring that he must be possessed by some evil spirit; while the surrounding relatives, the Turkish imans and dervises, as well as the Greek papas, stun the dying man with their cries.

"But premeditated poisonings are much more frequent than the accidental, and the native practitioners do not scruple to lend their ministry to these criminal transactions. According to the religious views of many Turks, crimes of this nature are not forbidden; as, in practising them, they are only endeavouring to be beforehand with their enemy, who is only watching for an opportunity to act in the very same manner with them: moreover, they consider that, if fate has not decreed the death of their enemy, any attempt to remove him will certainly miscarry, from some cause or other.

"But, if it is revolting to see an article of faith perverted in such a manner as to reconcile the mind to such crimes, how much more so is it to see them committed by Christians, who by no possible sophistry can reconcile them to themselves! But, unfortunately, it happens but too frequently that doctors, in the service of rich Turks, pachas, and the like, lend their aid to such misdeeds. It is, therefore, not at all advisable for a conscientious physician to enter into the service of a Turkish grandee, as his refusal to comply with such requests might not be unattended with personal danger." (P. 29.)

And, indeed, our author would seem, from his own account, to have proved, by personal experience, to what a pitch of degradation the noble profession of physic is reduced in that barbarous country, and to what dangers they are exposed who venture to thwart the prejudices or to resist the crimes of its inhabitants: he was at one time invited by a great officer to aid him in getting rid of his enemies, by administering poison to them; at another—and probably on account of refusing to do so—he was himself nearly poisoned, having found, one morning, something better than two drachms of sublimate at the bottom of his coffee-cup!

With such a state of medicine and of its professors as above described, it will hardly be expected that Turkey can boast of many of the medical institutions, whether for the communication of scientific or practical knowledge, which adorn more civilized nations. The present sultan, it is true, has established, at Constantinople, some kind of a school for the instruction of young men in medicine and surgery, and has, as we have already mentioned, caused to be printed for its use a sort of Medical Cyclopædia in the Turkish language; but this is the only thing of the kind in the empire. Neither are there any hospitals in Turkey for the reception of the sick. The only establishments of this kind that exist are lunatic asylums; and it is a curious fact that these are not only found in considerable numbers, but some of them are of great extent and even magnificence. This circumstance is explained by the universal belief among the Turks, that insane persons are either *possessed* or are objects of divine influence: in either case they are deemed sacred, and are venerated accordingly.

"In most large Turkish towns there are asylums for persons afflicted with diseases of the mind, called in Turkey *Timaristan,* which do the greatest honour to the heart and understanding of the people, as the management and order observed in them are much beyond the progress of civilization in other matters. These institutions are situated in the vicinity of the great mosques, and consist of convenient though not very clean cells, built round a court with piazzas in front. The Sulemanic Lunatic Asylum at Constantinople, connected with the mosque of the Sultan Suleiman, as well as that of the Sultan Selim at Adrianople, are of considerable extent. The first is a square building of hewn stone, in the Moorish style of architecture, about 180 feet in length, 160 in breadth, and one story high; the interior is divided into two courts, surrounded by a marble colonnade, which gives admittance to the wards. The roofs are a series of cupolas, intersected by arched vaults, and the second court is embellished with a beautiful fountain.

"Lunatic hospitals of this kind, but on a smaller scale, are to be met with in every part of the kingdom, containing a hundred and sometimes even a greater number of patients. Idiots and epileptic patients constitute the greatest number; of maniacs there are but few. As persons afflicted with mental diseases are in this country held sacred, they are treated with attention and humanity, and are, in fact, in a much better condition than the inmates of many asylums among the Christians. These Imarets, or asylums, however, are often very inadequately endowed, or their resources not properly managed, and consequently it is not easy to procure at all times sufficient food for the inmates. In these cases they depend on the compassion of their fellow believers, by whom they are irregularly fed. On no occasion does a Turk tease an idiot; he may smile at his follies, but he never leaves him without having first bestowed an alms on him." (P. 107.)

The inmates of these asylums are, as has been stated in the foregoing extract, chiefly idiots, while maniacal patients are rarely observed; and this is a circumstance well meriting the attention of the medical philosopher and the moralist. Here is a vast empire, in which one form of mental infirmity prevails, almost to the exclusion of other forms, so common and even more predominant than idiocy in other countries. The causes of this striking fact seem well explained by our author.

"It is proved by experience that, the higher the point to which the cultivation of the mind has attained in a country, the more frequent are the cases of mental derangement, and that with the rising morality of a nation homicide decreases, while suicide increases; in other words, that, generally, homicide and suicide stand in inverse ratio. Turkey offers the most convincing proofs of the correctness of this statement. In a country where the mind is sunk into an incurable lethargy, —where civilization is unable to rise out of the abject degradation into which it has fallen,—many of the usual causes of aberration of the mind must necessarily be wanting; such as excessive study, deep and prolonged reflection on any one subject, and such-like. Drunkenness, which with us is so frequently the cause of deranging the faculties of the mind, is unknown among the Turks, from their complete abstinence from spirituous liquors. By marrying when very young, they are enabled to satisfy the earliest desires for sexual connexion; and their imagination is never excited by obscene pictures or reading, nor their desires prematurely called forth by stimulating food; lastly, unrequited love, which has so baneful an effect on the sensorium, is unknown in the East, as well as romantic love in general. Here the virgin surrenders without a sacrifice, man conquers without a struggle, enjoys without delicacy, and passes suddenly from eager desire to satiety. Jealousy, the torture of man in Europe, is consequently unknown to the Turk; and if in cases of adultery, his vengeance falls with such dreadful weight upon his wife, as well as on her seducer,—he is not spurred on by jealousy, but by wrath against the contemner of his faith as well as of his person; for, according to his belief, whoever touches his wife dishonours his religion. The resignation enjoined to the Turk by his religion, as well as the fatalism in which he believes without the slightest scepticism, also contributes, in no small degree, to prevent the loss of property, dignity, or office, the disappointment of hopes, unsuccessful speculations, the death of a beloved person, &c., from producing mental derangement. Examples of Mussulmen being driven to despair, madness, or self-destruction, by misfortune, are certainly among the rarest occurrences. That, however, the reason of this is to be sought rather in the religion than in the low state of mental cultivation, is proved by the circumstance that, among the Christian inhabitants of the East, Greeks, Armenians, &c., who are but very little above the Mahometans in mental cultivation, diseases of the mind are comparatively much more frequent, and that cases of suicide, though but of rare occurrence, are however not quite unknown." (P. 99.)

If we can thus satisfactorily explain the comparative infrequency of furious madness or acute melancholia among the Turks, we are enabled, with at least equal certainty, to account for the great prevalence of idiocy among them; a prevalence which Dr. Oppenheim regards as greater than is to be met with in any other country. It is true that the apparent amount of prevalence is greater than the real, since we are told that a great many persons *feign* this disease; some, that they may enjoy the advantages attendant on the victims of so sacred a visitation; others, that they may escape the punishment of political offences, real or imputed, or that they may save their goods or persons from envy and revenge. Of the real idiots a certain portion are so from birth, but a good many are brought to this state through the use of opium and other narcotic poisons; particularly children, who are intentionally kept under the combined influence of such means, until the effect is produced.

As a general rule, the Turks, both men and women, enjoy remarkably good health, owing in a great measure to the absence of many of the arts and artificial modes of life which exert so great an influence over the inhabitants of more civilized countries. It is not, however, ascertained whether they are a long-lived race or not, owing to the total want of mortuary registers. Women begin to look old at an early period of life, owing, no doubt, partly at least, to the precocious development of some of the principal functions. "They have the catamenia about the tenth year, they marry in the twelfth, soon become mothers, have many children, cease to menstruate at thirty, and speedily fade into old age." (P. 54.) Their labours are in general remarkably easy, insomuch that even women of the upper ranks are on their feet on the second day, and on the third leave their chamber to take a bath. The mothers invariably nurse their children, but the ladies consign this task to a wet-nurse during the night. It is the law of Mahomet that the child should be suckled two years, if it will take the breast so long; but this term may be abridged with permission of the husband. During the period of nursing, sexual intercourse is strictly forbidden by the Koran. Abortion, intentionally procured, is extremely common, particularly among the higher ranks: it is not regarded as criminal by the Mohammedan law until the fifth month of pregnancy, at which period the fœtus is considered to become possessed of life.

We shall conclude this review of the State of Medicine in Turkey by noticing such of the diseases, whether medical or surgical, as present, in the account given of them by the author, anything interesting, either as regards pathology or practice.

Vaccination was many years since introduced into Turkey by Dr. De Carro, of Vienna, but has as yet made but little progress in the interior of the kingdom. The present sultan has had his own children vaccinated by Dr. Auban, a French physician. Inoculation of the smallpox, first practised in Turkey, is in much more general use: it is chiefly in the hands of the women, who operate by making some superficial incisions with a razor, and then placing in these either a thread impregnated with the variolous fluid, or some of the pulverised scabs.

Scarlatina appeared to Dr. Oppenheim very prevalent and very fatal in Turkey; but the most interesting circumstance respecting it stated by him, is what he regards as a marked instance of the prophylactic powers of belladonna on the large scale.

"The disease had also broken out among the troops in Monastir, where no separation of the unhealthy from the healthy was practicable; several soldiers had been carried off, and the disease had even snatched a victim from the grand vizir's harem. The vizir, who loved his troops as his children, became alarmed; when I thought it my duty to make him acquainted with our preventive measures. He issued an order to his soldiers to take carefully a medicine that would be prepared for them; and, as he set the example by taking it himself from the hands of his physician, they obeyed with less reluctance. As I had to fear that the counting of the drops would not be properly attended to, I preferred the form of pills, in spite of the opposition of the apothecaries, who for a whole week were engaged in nothing else than making pills, although only two battalions (1,200 men) of regular troops were at that time in the town. I directed one pound of extract of liquorice to be mixed with thirty-six grains of extract of belladonna, and the mass to be made into two-grain pills, of which each soldier was to take five morning and evening. I entertained the hope that this dose, though smaller than usually prescribed, would have the desired effect with men who had never taken any medicine, and who all were between the ages of fifteen and twenty-five; and the result answered my expectation; for, although the epidemic continued to rage in the city, and the soldiers were quartered on the Christian and Jewish part of the population, only twelve of all that had taken the belladonna fell sick, of which, however, one-half died. In ten days previously to the administering of the medicine more than forty cases of sickness had been entered on the sick-list." (P. 57.)

Measles were never observed by our author, nor could he find, from his medical friends, that they prevailed in the country. Worms are of frequent occurrence among children, and are cured by many popular remedies; among others, the powdered kernel of the wild apricot. Dr. O. met with six cases of Guinea-worm. Hydrocephalus is said to be common; but internal scrofula and rickets are decidedly rarer than among European children; a circumstance which our author attributes to the use of a wholesome diet, chiefly rice, and the enjoyment of a purer air.

In all nervous and convulsive diseases, the ancient remedy, *Bezoar-stone*, enjoys the highest reputation, and is indeed in such request that a profitable trade is driven in manufacturing artificial substitutes for it.

Spitting on the affected part and also a sort of animal-magnetism are favorite and effectual remedies. The manipulations of an Asian Dervish, noticed in the following account, would not discredit a disciple of Mesmer himself.

"The patient was suffering from headach, and sat in the Turkish fashion on the ground. The operator knelt before him, stroked his forehead with the thumbs of both hands from within outwards, then drew the skin in folds or wrinkles in an opposite direction, pressing it at the same time forcibly, repeated a prayer, spit two or three times on the ground, then on the affected part, and at last rose up, convinced, as well as the patient, that the complaint would speedily vanish." (P. 70.)

Intermittent fever, in all its forms, is extremely common, and is by the native doctors attributed to the presence of an evil spirit, and attempted to be cured by exorcism; the same opinions and practice hold with regard to gout and rheumatism. Quinine is however used by the Greek and Turkish doctors; but can hardly be obtained pure.

The following account of a disease endemic in Turkey and which Dr. Oppenheim considers as having hitherto escaped the observation of European physicians, is curious. We agree with him in regarding it as unknown in northern Europe at least. He proposes to call it *Rheumatische Knollenkrankheit* (literally "the rheumatic knobby disease"); but we have great doubts of its rheumatic nature. It reminds us somewhat of a peculiar acute disease of the lymphatics of the extremities prevalent in the West Indies, and which appears, in some cases at least, to be analogous to, if not the actual precursor of the elephantiasis or Barbadoes leg.

"I noticed a particular endemic disease, which I believe has hitherto escaped observation, and, as it is probably closely related to rheumatism, I feel inclined to give it the foregoing name. Round hard tumours will frequently rise in a single night, though the patient may have retired to rest in perfect health, sometimes without any ascribable cause and at others after suppressed perspiration, or cold caught on sudden changes of temperature; they always make their appearance between two joints, or at least at some distance from the joint, and constantly on the flexor side of the limb; the tumours, as before mentioned, are round, hard, the extreme circumference undefined, not easily moved, and very painful when touched; the skin remains unchanged and without inflammation, and the temperature is not heightened; they vary in dimension from that of a hazel-nut to that of the fist, and attain this size in the course of a few hours; they then remain stationary. These tumours more frequently occur in the upper than in the lower extremities, and again more frequently in the forearm and leg than in the upper portions of the same limbs; very frequently they appear in the palm of the hand, which then swells up to the form of a ball, also in the sole of the foot, and on the fingers and toes: sometimes only one tumour appears at the same time, but more commonly several.

"Under this disease the general health is unchanged; all the functions are regularly performed; digestion alone seems in a small degree disturbed, the tongue being loaded, &c. This disease offers no premonitory symptoms. The cellular texture immediately surrounding the muscles appears to be the seat of it, in which a local, abnormal, excited or more probably suppressed activity of the lymphatic vessels, produces an exudation. Young, strong, and robust persons are more subject to this disease than the aged, weakly, and cachectic; men also are more liable than women; children are rarely or never afflicted with it.

"Persons not accustomed to the climate are said to be very liable to it. The prognosis is very favorable if assistance be speedily called in; but if neglected, these tumours will continue during the whole life of the patient, and by their pressure occasion pain at every motion of the limb; stiffness and incapacity of bending the joint follows, and, at last, total inactivity and continued pressure bring on decay.

"The remedy used by the natives is as simple as it is effective, and consists in uninterruptedly rubbing and kneading the part from below upwards, in semicircular movements, either with the first joint of both thumbs or with the palm of the hand. The rubbing is continued in spite of the suffering and complaining of the patient till the tumour has completely disappeared, and is frequently continued for four or six hours and sometimes longer. The operation is most effective when performed in the Turkish sweating-bath. A repetition of the operation is not esteemed necessary, though returns of the disease are not unfrequent. In almost every place there are some persons, most frequently women, who are exclusively engaged in this rubbing process. As a matter of course, mystic incantations are not omitted in the operation. In old standing cases, no remedies whatever are attempted, although very probably strong stimulating means would effect the reabsorption." (P. 74.)

In an account of the state of health and disease in Turkey, it would be impossible to overlook the well-known habit of opium-eating and its effects. Drinking wine being forbidden by the laws of the prophet, the habit of drunkenness is rare in Turkey; but as the culprit is equally guilty and equally obnoxious to punishment, whether he drinks little or much, it commonly happens, our author says, that the person who once commits a debauch, becomes a perfect drunkard. And although not forbidden, like wine-drinking, by the Koran, opium-eating almost invariably leads to the same extravagant excess and melancholy results. Once begun, it is hardly ever relinquished; once a *Theriaki* always a *Theriaki*.

"The causes leading to the use of opium are many, and among them may be reckoned the following: long continued diarrhœa, as a remedy for which opium is used in the first instance, and its use afterwards continued from habit; chronic coughs, in which opium is also used as a popular medicine; habitual drunkards also frequently have recourse to opium as a new stimulus, after they have abjured wine in some fit of repentance. Persons holding high offices or dignities in the state also have recourse to opium, when the preservation of their character forbids them the use of wine: some very strict believers also take opium as a restorative in cases of great exertion, as the *Tatars* (couriers), who travel with astonishing celerity.

"Opium eaters generally begin with doses of from half a grain to two grains, and gradually increase the quantity till it amounts to two drachms and sometimes more a day; they usually take the opium in pills, but avoid drinking any water after having swallowed them, as this is said to produce violent cholic: to make it more palatable, it is sometimes mixed with syrups or thickened juices; but in this form it is less intoxicating and resembles mead; it is then taken with a spoon or is dried in small cakes, with the words '*Mash Allah*,' 'the work of God,' imprinted on them.

"The effect of the opium manifests itself one or two hours after it has been taken, and lasts for four or six hours, according to the dose taken and the idiosyncracy of the subject. In persons accustomed to take it, it produces a high degree of animation, which the *Theriaki* (opium-eaters) represent as the acme of happiness.

"The habitual opium-eater is instantly recognized by his appearance. A total attenuation of body, a withered, yellow countenance, a lame gait, a bending of the spine, frequently to such a degree as to assume a circular form, and glossy, deep sunken eyes, betray him at the first glance. The digestive organs are in the highest degree disturbed, the sufferer eats scarcely any thing and has hardly one evacuation in a week: his mental and bodily powers are destroyed,—he is impotent. By degrees, as the habit becomes more confirmed, his strength continues decreasing, the craving for the stimulus becomes ever greater, and, to produce the desired effect, the dose must constantly be augmented.

"When the dose of two or three drachms a day no longer produces the beatific intoxication so eagerly sought by the Opiophagi, they mix the opium with [corrosive] *sublimate,* increasing the quantity till it reaches to ten grains a day; it then acts as a stimulant.

"After long indulgence the opium eater becomes subject to nervous or neuralgic pains, to which opium itself brings no relief. These people seldom attain the age of forty, if they have begun to use opium at an early age. The fasts in the month of Ramasan are for them fraught with the most dreadful tortures, as during the whole of that month they are not allowed to take any thing during the day. It is said that, to assuage their sufferings, they swallow, before the morning prayer, besides the usual dose, a certain number of other doses, each wrapped up in its particular paper, having previously calculated the time when each envelope shall be unfolded and allow the pill to produce the effects of their usual allowance. When this baneful habit has become confirmed, it is almost impossible to break it off; the torments of the opium-eater, when deprived of this stimulant, are as dreadful as his bliss is complete when he has taken it; to him night brings the torments of hell, day the bliss of paradise. Those who do make the attempt to discontinue the use of opium, usually mix it with wax, and daily diminishing the quantity of the opium, the pill at last contains nothing but wax." (P. 93.)

We shall conclude this article with a few remarks on the state of surgery in Turkey, which Dr. Oppenheim informs us is even in a still lower state than that of medicine, and the chief cause of which he justly refers to the total unacquaintance of the practitioners with anatomical knowledge. And here, as in almost every thing else, the Mahommedan religion interferes most injuriously. Not only is dissection forbidden by the Koran, as we have already said, but almost all the subjects of surgery are as it were *tabooed* by the debasing superstition which it enforces. Every injured person, whether with open wounds or not, is unclean, and all unnecessary spilling of blood is rigidly forbidden. When to these causes we add the considerations that to the Turks the life of any man is of small value, while to all death is a blessing, we need not be surprised to learn that operative surgery, at least, is at the lowest possible ebb in this wretched country.

Notwithstanding their prejudices and the dogmas of the Koran, still the Turkish surgeons do draw blood, sometimes even more plentifully than we ourselves should be inclined to do as a remedy, and also perform bloody operations. Scarification is a frequent and favorite remedy in many diseases, and is performed in the primitive fashion, originally common among most uncivilized nations, and still not quite obsolete, we believe, in the highlands and islands of Scotland, viz. by making incision with a razor and using the mouth and a sheep's horn as an exhausting means for extracting the blood. Venesection is also in frequent use, and is performed by means of a round-pointed lancet contained in a sheath, from which it is projected, by means of a bow or spring, to a certain depth into the vein.

"Many so-called surgeons confine their practice to the treatment of one particular disease or to one particular kind of operation: there are consequently, as there used to be in Europe, bonesetters, operators for rupture, cataract, stone, &c. and who frequently, in spite of their ignorance, enjoy considerable fame and confidence. The eye-doctors are principally Persians and Arabians; the operators for rupture and stone, as well as the bone-setters, are for the most part *Moreots* and *Zagoreots*. The method of performing these operations is generally inherited from father to son, and is consequently an heir loom of a few families. On this account, they also conceal their mode of treatment as much as possible, and allow neither the initiated nor the uninitiated to be present at their operations; even the nearest relatives are dismissed from the apartment, and the operator remains alone with the patient, seldom admitting an assistant. Notwithstanding the express command of the Grand Vizier to the surgeons, to admit me to all their operations, I succeeded but in very few instances to be present: every kind of excuse to avoid complying with this order was put in practice, and even the patients were induced to request that I should not be admitted to the operation. The most ostentatious of all these operators were the bone-setters, who unblushingly asserted that they could cure any fracture, however complicated and however much the bones might be splintered; and that the limb would suffer injury neither in form nor utility, and on this ground they claim the superiority of their own surgery over the European, as their certainty of healing the limb renders amputation unnecessary, to which we are obliged to have recourse when unable to heal a fracture.

"Exaggerated, boasting, and bombastic as these assertions are, it is however not to be denied that, with all their ignorance, they treat fractures with very great success. Their method of placing the limb in its proper position, and keeping it in that position during the cure, by means of a gypsum case or envelope, is well known." (P. 114.)

"The treatment of luxations, the most difficult part of surgery, and which requires the most accurate knowledge of anatomy, is engrossed principally by the bone-setters and some female practitioners. Several old luxations of the shoulder and elbow which I have had to treat, gave me no high opinion of the skill of these people." (P. 117.)

"Cases of hernia are common, not only among the lower order of the Turks, but also among the higher classes, and often prove fatal from bad treatment or neglect. The early age at which they begin to ride, and the constant practice of this exercise, on ill-constructed saddles and rough roads, may be assigned as the principal cause of rupture among them. In no part of the empire are there any trusses manufactured: some of the grandees procure them from Vienna, France, or Italy, but, after long use, repairs, and the alterations they are made to undergo, they resemble any thing as much as what they were originally intended for. The operation for strangulated hernia is unknown to the Turkish surgeons, but they profess to cure radically such cases as are not strangulated, and this they effect by ligature and the actual cautery.

"I had an opportunity of seeing this operation performed in Jenitschar (Larissa), by a surgeon from Sagor named Michalaki. The patient was a powerful man of rather more than forty, who had suffered for a considerable time from an inguinal rupture, which by its increase in size had now become very inconvenient in riding. After the operator had convinced himself that the rupture admitted of being pushed back, the patient was secured, with his arms crossed, to a board, and this so placed that the feet were directed upwards and the head down. With one hand the operator held back the rupture, and with the other he made with a razor an incision of about three inches in length, extending from about one inch above to two inches below Poupart's ligament. He then, as he expressed himself, got sight of a bladder (the hernial sac); this he drew forward with both hands as far as he could, he then passed a strong silken thread under this sac close to the inguinal ring, and having secured it, cut through the sac below the ligature; but he had included the spermatic cord in the ligature, so that the testicle must afterwards have decayed." (P. 117-119.)

"The stone is of frequent occurrence in some provinces of Turkey, namely, Macedonia, Epirus, Thessaly, &c., and in some families is even an hereditary disease. The method of the Turkish stone-cutters is as follows: The sick person suspected of having a stone in the bladder, is examined by the operator who introduces a finger into the anus, and feels about the perinæum with the other. If in this manner he can find a stone, he determines on the operation. His method is the old Celsian. He knows of neither sound, probe, nor forceps. His whole stock of instruments consists of a razor, for the incision, and a blunt indented hook, which is used when the stone is too large for the aperture or presents itself in such a manner that it cannot be removed by pressure or by the finger. When the patient has been secured and his legs held up in such a position that the perinæum is distended, the operator introduces his forefinger into the anus, presses the stone downwards, and makes an incision on the part where it most projects, consequently without any consideration whether it be on the right or the left side of the anus; the incision is made at one cut through the skin, fat, cellular texture, and the coats of the bladder, the operator regulating the length of the wound by the size of the stone, which after the incision has been made springs forward, on the pressure of the finger in the anus, just as is the case in cataracts when the incision of the cornea is made. Should the stone, however, present itself unfavorably for extraction or be too large for the orifice of the incision, the operator has then recourse to the above-mentioned hook indented on the inside, and with this he endeavours to bring the most favorable diameter of the stone to the aperture, and then extracts it with more or less violence. But it sometimes occurs that even with the assistance of the hook, the operator fails in extracting the stone, and that then the operation has been undertaken in vain: in this case the patient is left to his fate. After the stone has been extracted, the wound is stopped with cotton, on which some mild salve has been spread, and the business of the operator is ended. No after treatment takes place, nor are any means used to prevent inflammation or to remove it, if it has already taken place; no particular rule of diet is followed; on the contrary, the patient is denied the rest of which he is so much in need of, and for the first twenty-four hours after the operation he is prevented from sleeping by the uproar and noise of the guests aided by discordant music; and he is even awaked, if, in spite of all this, sleep should prevail. Whoever happens to be in the sick chamber at sun-set is not allowed to leave it before sun-rise, nor are visitors denied admittance at a later hour. The recovery is usually complete in from ten days to a fortnight, and according to the assertion of the operator two-thirds of the persons operated on recover. Of the causes of death in such cases my informant had never thought, had no idea of inflammation, and never knew death from either internal or external hemorrhage. He showed me several stones of considerable size, which he had removed by the operation, and the former possessors of which were still alive." (P. 121-123.)

With these extracts we conclude this highly interesting work; and we cannot take our leave of it without expressing our opinion, in strong terms, of the intelligence, industry, and discrimination displayed by its learned author in the collecting and arranging his materials in their present form. The contemplation of such a mass of ignorance, superstition, and immorality as is contained in the preceding pages cannot fail to reconcile us to much that is irksome in our own lot, to make us grateful and proud that it is our good fortune to know and to practise medicine as it exists among ourselves, and to cheer us with the hope that, since the labours of our predecessors in the more civilized nations of Europe have already advanced it to a state of comparative perfection when contrasted with that in which it exists in Turkey, the exertions of our successors may go still further, and with more rapid strides, to bring it yet nearer to the point of scientific certainty and practical precision, which every benevolent mind must so ardently desire.

*Note.* We have just noticed in one of the German Journals (Zeitschrift, &c. Feb. 1837,) that Chomel's work on Pathology has been translated into Turkish by command of the sultan, and printed for the use of the students of the medical school of Constantinople at the imperial press. The translator is Osman-Effendi, son of one of the most distinguished Ulemas. The price of the volume is eight piastres, (189 groschen.)

